# Postoperative serum changes in calcium, phosphorus, iPTH, CRP, IL-6, and TNF-p levels in patients with secondary hyperparathyroidism

**DOI:** 10.5937/jomb0-55372

**Published:** 2025-08-21

**Authors:** Wenjing Feng, Runtao Xu, Jinghui Mu, Li Jie, Mingjing Cheng

**Affiliations:** 1 Third Hospital of Hebei Medical University, General Surgery, Shijiazhuang City, Hebei Province, China

**Keywords:** secondary hyperparathyroidism, fast-track surgery, food structure intervention, nutritional status, immune response, calcium-phosphorus metabolism, postoperative recovery, sekundarni hiperparatiroidizam, brza hirurgija, intervencija u strukturi hrane, nutritivni status, imunološki odgovor, metabolizam kalcijum-fosfora, postoperative oporavak

## Abstract

**Background:**

This study investigates the impact of food structure intervention based on the fast-track surgery (FTS) concept on the postoperative nutritional status, immune response, and recovery outcomes of patients with secondary hyperparathyroidism (SHPT). Specifically, it explores changes in serum calcium and phosphorus levels, immune-reactive parathyroid hormone (iPTH), inflammatory markers (CRf) IL-6, TNF-P), and rehabilitation outcomes following surgical intervention.

**Methods:**

Fifty SHPT patients who underwent surgery at the Third Hospital of Hebei Medical University were randomly divided into two groups: a control group (CG) receiving conventional nursing care and an observation group (OG) receiving food structure intervention based on the FTS concept. Preand postoperative comparisons were made for nutritional indicators (hemoglobin, albumin), biochemical markers (calcium, phosphorus, calcium-phosphorus product, iPTH), inflammatory markers (CRF) IL-6, TNF-P), pain levels (Visual Analog Scale), muscle strength (MRC scale), and postoperative complications.

**Results:**

The OG showed significantly improved nutritional status, with higher hemoglobin and albumin levels compared to the CG. Additionally, blood calcium levels and calcium-phosphorus product were significantly elevated, while blood phosphorus and iPTH levels were reduced in the OG. Inflammatory markers (CRf) IL-6, TNF-P) were significantly lower in the OG. Pain scores (VAS) were lower, muscle strength (MRC) was higher, and the incidence of complications was significantly reduced in the OG compared to the CG.

**Conclusions:**

Food structure intervention based on the FTS concept enhances nutritional status, improves mineral metabolism, reduces postoperative inflammation, and accelerates recovery in SHPT patients. This study supports the implementation of FTS principles in perioperative care to improve outcomes and reduce complications for SHPT patients, offering valuable insights for optimising clinical management and nursing practices in this patient population.

## Abstract (sr)

**Uvod:**

Ova studija istražuje uticaj intervencije u strukturi hrane zasnovane na konceptu brze hirurgije (FTS) na post-operative nutritivni status, imunološki odgovor i ishode oporavka pacijenata sa sekundarnim hiperparatiroidizmom (SHPT). Konkretno, istražuju se promene u nivoima kalcijuma i fosfora u serumu, imunoreaktivnog paratiroidnog hormona (iPTH), inflamatornih markera (CRf) IL-6, TNF-P) i rehabilitacionih ishoda nakon hirurške intervencije.

**Metode:**

Pedeset pacijenata sa SHPT koji su podvrgnuti operaciji u Trećoj bolnici Hebei medicinskog univerziteta nasumično je podeljeno u dve grupe: kontrolnu grupu (CG) koja je primala konvencionalnu negu i grupu za posmatranje (OG) koja je primala intervenciju u strukturi hrane zasnovanu na konceptu FTS. Upoređivani su preoperativni i postoperativni podaci za nutritivne indikatore (hemoglobin, albumin), biohemijske markere (kalcijum, fosfor, proizvod kalcijum-fosfor, iPTH), inflamatorne markere (CRF) IL-6, TNF-P), nivoe bola (vizuelna analogna skala), mišićnu snagu (MRC skala) i postoperativne komplikacije.

**Rezultati:**

OG je pokazala značajno poboljšanje nutritivnog statusa, sa višim nivoima hemoglobina i albumina u poređenju sa CG. Takođe, nivoi kalcijuma u krvi i proizvod kalcijum-fosfor bili su značajno povećani, dok su nivoi fosfora u krvi i iPTH bili smanjeni u OG. Inflamatorni marked (CRF) IL-6, TNF-P) bili su značajno niži u OG. Nivoi bola (VAS) bili su niži, mišićna snaga (MRC) visa, a učestalost komplikacija značajno smanjena u OG u poređenju sa CG.

**Zaključak:**

Intervencija u strukturi hrane zasnovana na konceptu FTS poboljšava nutritivni status, poboljšava metabolizam minerala, smanjuje postoperativnu inflamaciju i ubrzava oporavak kod pacijenata sa SHPT. Ova studija podržava primenu principa FTS u perioperativnoj nezi kako bi se poboljšali ishodi i smanjile komplikacije kod pacijenata sa SHPT, nudeći dragocene uvide za optimizaciju kliničkog upravljanja i praksi nege kod ove populacije pacijenata.

## Introduction

The parathyroid glands are essential endocrine organs that play a pivotal role in regulating calcium and phosphorus metabolism in the body. By secreting parathyroid hormone (PTH), these glands help maintain calcium homeostasis, a process critical for numerous physiological functions, including bone health, muscle function, and nerve signalling [1] [2]. However, when there is a disruption in the body's calcium-phosphorus balance, such as in cases of chronic kidney disease (CKD) or other related disorders, the parathyroid gland compensates by increasing PTH secretion, a condition known as secondary hyperparathyroidism (SHPT). This overproduction of PTH leads to elevated blood calcium levels while phosphorus levels decline, causing disturbances in mineral metabolism that may result in significant health complications [3] [4].

SHPT is particularly prevalent in patients with CKD undergoing maintenance hemodialysis, where long-term renal dysfunction impairs the body's ability to excrete phosphorus and activate vitamin D, both of which are crucial for regulating calcium levels [5] [6]. The clinical manifestations of SHPT, including bone demineralisation and vascular calcification, contribute to the high morbidity and mortality rates seen in these patients, particularly from cardiovascular diseases [7] [8]. As such, managing SHPT is vital in improving the health outcomes of patients with advanced kidney disease.

Total parathyroidectomy (PTX) combined with autologous parathyroid transplantation has become an established treatment option for patients with severe SHPT, especially when pharmacological treatments fail [9]. However, surgical interventions inherently carry risks such as postoperative pain, delayed recovery, and an increased likelihood of complications, including infections and pressure ulcers, all of which can compromise the overall prognosis and quality of life of the patient [10]. Given the traumatic nature of surgery, the perioperative care process plays a crucial role in mitigating these risks and accelerating recovery.

Fast-track surgery (FTS) has gained traction in recent years as an innovative approach to optimising perioperative care. FTS focuses on minimising surgical stress, reducing the duration of hospital stays, and improving recovery outcomes by implementing a range of evidence-based interventions, including enhanced pain management, early mobilisation, and optimised nutritional strategies [11] [12]. Among the various strategies, nutrition has emerged as a key factor in supporting the body's healing process postsurgery. Ensuring that patients receive adequate and appropriate nutritional support is crucial for maintaining immune function, reducing inflammation, and promoting tissue repair [13].

An often overlooked yet significant aspect of postoperative care is the role of food structure interventions, which involve tailored dietary modifications to address the specific nutritional needs of patients. A well-balanced, nutrient-dense diet can improve the nutritional status of surgical patients, reduce immune stress, and lower the risk of complications such as infection or delayed wound healing [14] [15]. In the context of SHPT, where calcium, phosphorus, and vitamin D imbalances are common, a specialised food structure intervention is particularly important in managing mineral metabolism and enhancing recovery.

The current study investigates the effects of food structure intervention based on the FTS concept on postoperative nutritional status, immune stress, and rehabilitation outcomes in SHPT patients. By comparing the clinical outcomes of patients who received conventional nursing care with those who underwent a more integrated care approach involving FTS principles and targeted dietary interventions, this research aims to elucidate the potential benefits of this model in improving postoperative recovery and long-term health outcomes. Specifically, we will evaluate changes in serum calcium and phosphorus levels, immune reactive parathyroid hormone (iPTH) levels, inflammatory markers such as C-reactive protein (CRP), interleukin-6 (IL-6), and tumour necrosis factor-beta (TNF-β), as well as the overall nutritional status and recovery trajectory of patients.

By enhancing our understanding of how FTS and food structure interventions can support the recovery of SHPT patients, this study hopes to provide valuable insights into improving perioperative care protocols and optimising the management of this challenging patient population.

## Materials and methods

### General data

Fifty SHPT patients who underwent surgery in our hospital from January 2024 to December 2024 received selection and random division into the control group (CG) and observation group (OG), with 25 cases each. CG: 16 males and 9 females; mean age was 47.17 ± 5.15 years old. OG: 14 males and 11 females; mean age was 46.98 ± 5.20 years old. Patients and their family members in this research signed informed consent after understanding specific research content and process.

### Inclusion and exclusion criteria

Inclusion criteria: (1) Patients needed to maintain hemodialysis; (2) symptoms such as bone pain, osteoporosis, and skin itching were clinical manifestations; (3) imaging examination revealed enlargement of one or more parathyroid glands in patients with a diameter greater than 1 cm or a maximum volume exceeding 300 mm3; (4) met diagnostic criteria for SHPT; (5) recommended and treated with surgery after diagnosis by medical staff. Exclusion criteria: (1) Concomitant with severe important organ diseases; (2) according to diagnosis by the anaesthesia department, there was a huge risk during the perioperative period; (3) those with coagulation dysfunction; (4) those with severe skeletal deformities; (5) those with cognitive impairment or low compliance due to mental illness.

### Methods

The CG received conventional nursing for patients, including preoperative examination, surgical preparation, preoperative dietary guidance, health education, vital sign monitoring, and postoperative position nursing.

The OG received food structure intervention based on the FTS concept for patients. 1) Psychological intervention. Nursing staff should provide targeted counselling to patients based on their psychological burden, emphasise a stable mindset and surgical stress relationship, and guide patients in emotional regulation methods, encouraging and comforting patients. 2) Preoperative guidance. Nursing staff should guide patients in practising surgical positions. 3) Preoperative preparation. Nursing staff should be prepared to prevent patients from falling. Nursing staff should provide patients with a higher-quality ward environment, hang anti-fall warning signs in prominent positions in wards, guide patient families to pay attention to patient's condition and do a good job in safety nursing. When carrying out clinical nursing work, nursing staff should ensure gentle and slow movements to avoid the occurrence of patients' fractures caused by external forces. Nursing staff should guide patients to fast solids for 6h, liquids for 2h before surgery, and drink water 2h before surgery to alleviate their stress response. Antibiotics should be applied 0.5h before surgery. 4) Intraoperative nursing. Nursing staff should monitor patients' body temperature and strengthen temperature maintenance nursing. The liquid applied during surgery should receive heating, and the operating room temperature should be adjusted reasonably. 5) Postoperative nursing. Nursing staff should assist patients lying supine after surgery, monitor their blood pressure and vital signs, and maintain smooth drainage. Additionally, nursing staff need to check patients' blood calcium levels regularly and whether there is numbness in the face, hands, and feet. Once such abnormal conditions occur, medical personnel should be notified immediately for timely treatment. Nursing staff should provide pain relief to patients based on their pain level. 6) Discharge guidance. Nursing staff should know patients' lifestyle habits and guide them on a reasonable diet and exercise. Also, nursing staff should communicate with family members and instruct them to supervise patients' daily lives and other aspects. 7) Food structure intervention: A nursing staff should develop a food structure table based on the patient's physical and mental characteristics and dietary habits. Nursing staff should make food structure charts into cards and distribute them to patients. b. Nursing staff should guide patients to eat iodine-free and high protein, high calorie, and high vitamin diets. Appropriate calcium supply refers to the supply of foods with high calcium content. Patients should consume a duck egg and 200 g of tofu daily. An iodine-free diet refers to the intake of non-iodised salt. Patients should avoid foods high in iodine with a daily salt intake of less than 6 g/d. A high vitamin diet refers to eating more fresh vegetables and fruits, with a variety of 5 per day, vegetables 500 g daily, and fruits around 300 g daily. Colourful vegetables should be chosen more frequently. c. A food structure adjustment nursing team should receive establishment, including head nurses, clinical nutritionists, charge nurses, etc. Nursing staff should strengthen dietary education for patients, make them aware of the importance of food structure intervention, enhance their compliance with dietary nursing, avoid overeating, and have more meals daily but less food at each. d. Nursing staff should check the implementation of dietary guidance on patients daily and adjust dietary content based on patient feedback.

### Observation indicators

(1) Nutritional indicators: The haemoglobin (HGB) and albumin (ALB) levels between both groups before and after intervention received comparison. The 6 mL of fasting venous blood received extraction from patients. A portion of blood samples received centrifugation (3500 r/min, 15 min), serum received collection, and serum ALB level received detection with a fully automated biochemical analyser. The other portion of blood was measured for HGB level with a fully automated blood cell analyser.

(2) Biochemical indicators: The blood calcium and blood phosphorus levels, calcium-phosphorus product, and immune reactive parathyroid hormone (iPTH) level between both groups before and after intervention received comparison. The blood sample collection and serum preparation methods were the same as above. The blood calcium and phosphorus levels, calcium-phosphorus product, and iPTH level were detected with a fully automated biochemical analyser.

(3) Inflammatory indicators: The C-reactive protein (CRP), interleukin-1 (IL-1), and tumour necrosis factor-β (TNF-β) between both groups before and after intervention received comparison. The blood sample collection and serum preparation methods were the same as above. The serum CRP IL-6, and TNF-β levels received detection through enzyme-linked immunosorbent assay (ELISA).

(4) Prognostic indicators: The postoperative pain and muscle strength between both groups received comparison. The pain was evaluated using the Visual Analog Scale (VAS) [16], with a score range of 0-10 points. The higher the scores, the more pronounced the patients' pain is. The muscle strength was evaluated using the MRC muscle strength grading scale [17]. A score of 0-48 points indicates weakness in the limbs of patients, while a score of 48-60 points indicates normal muscle strength in the limbs of patients.

(5) Complications. The postoperative complications such as hypocalcemia convulsions, pressure ulcers, difficulty expectoration due to excessive phlegm, and nausea and vomiting in both groups received recording.

### Statistical analysis

The data analysis was conducted using SPSS 27.0 software. Counting data received expression as percentage, followed by χ^2^ test for intergroup comparisons. The measurement data received is represented by (x̄ ± s), followed by a t-test for intergroup comparisons. The difference was statistically significant with P<0.05.

## Results

### Comparison of general data between both groups

No statistical significance in general data was exhibited between both groups (P>0.05; Table 1), indicating comparability.

**Table 1:** General data in both groups.

Groups	N	Gender [n (%)]		Age (years)
		Male	Female	
CG	25	16 (64.0)	9 (36.0)	47.17±5.15
OG	25	14 (56.0)	11 (44.0)	46.98±5.20
χ^2^/t		1.333		0.109
P		0.248		0.914

### Comparison of nutritional indicators between both groups

**Figure 1:**
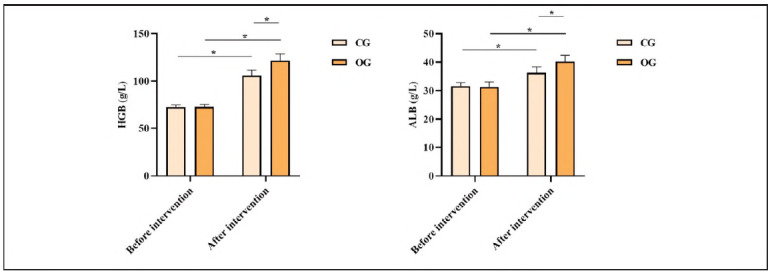
Nutritional indicators in both groups.<br>Note: *P<0.05, compared with before intervention

Before the intervention, neither group exhibited statistical significance in HGB and ALB levels (P>0.05). After the intervention, HGB and ALB levels in both groups exhibited elevation relative to those before the intervention. HGB and ALB levels in OG exhibited elevation relative to CG, indicating statistical significance (P<0.05; Figure 1).

### Comparison of biochemical indicators between both groups

**Figure 2:**
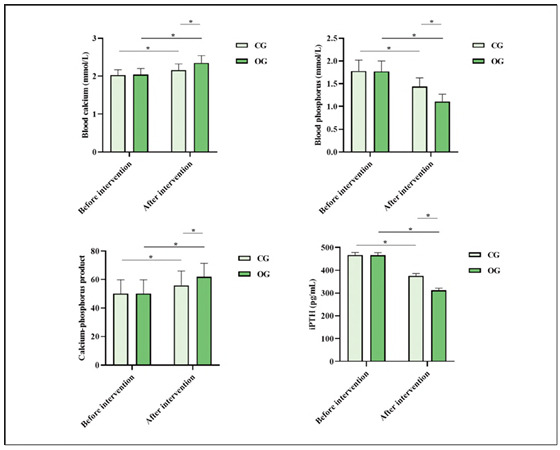
Biochemical indicators in both groups.<br>Note: *P<0.05, compared with before intervention

Before the intervention, both groups exhibited no statistical significance in blood calcium and blood phosphorus levels, calcium-phosphorus product, and iPTH level (P>0.05). After the intervention, blood calcium levels and calcium-phosphorus product in both groups exhibited elevation relative to those before intervention, while blood phosphorus and iPTH levels exhibited depletion relative to those before intervention. Meanwhile, blood calcium level and calcium-phosphorus product in OG exhibited elevation relative to those in CG, while blood phosphorus and iPTH levels in OG exhibited depletion relative to those in CG, indicating statistical significance(P<0.05; Figure 2).

### Comparison of inflammatory indicators between both groups

**Figure 3:**
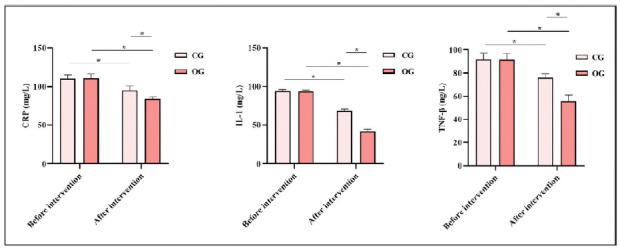
Inflammatory indicators in both groups.<br>Note: *P<0.05, compared with before intervention.

Before intervention, neither group exhibited statistical significance in CRP IL-1, or TNF-β levels (P>0.05). After the intervention, CRP IL-1, and TNF-β levels in both groups exhibited depletion relative to those before the intervention, and CRP, IL-1, and TNF-β levels in OG exhibited depletion relative to those in CG, indicating statistical significance (P<0.05; Figure 3).

### Comparison of prognostic indicators between both groups

**Figure 4:**
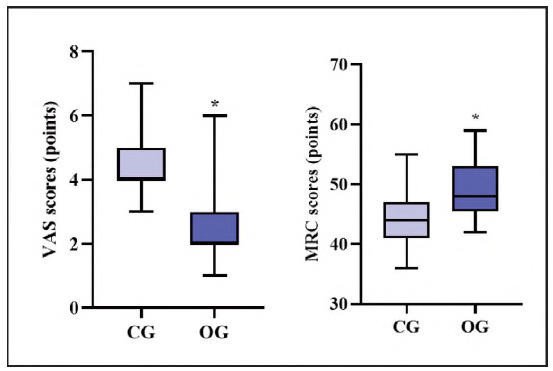
Prognostic indicators in both groups.<br>Note: *P<0.05, compared with CG.

The VAS scores in OG exhibited depletion relative to those in CG, while MRC scores in OG exhibited elevation relative to CG, indicating statistical significance (P<0.05; Figure 4).

### Comparison of incidence of complications between both groups

The incidence of complications in OG exhibited depletion relative to that in CG, indicating statistical significance (P<0.05; Table 2).

**Table 2:** Incidence of complications in both groups.

Groups	N	Postoperative<br>hypocalcemia convulsions	Pressure<br>sores	Difficult expectoration<br>due to excessive phlegm	Nausea and<br>vomiting	Total complication<br>rate [n (%)]
CG	25	1	3	1	1	6 (24.0)
OG	25	0	1	0	1	2 (8.0)
χ^2^						9.524
P						0.002

## Discussion

Dialysis is a key technology for treating CKD, and with the advancement of dialysis technology, the survival time of CKD patients is markedly prolonged; with prolongation of dialysis treatment time, almost all patients will experience a marked decline in vitamin D level and hypocalcemia and/or hyperphosphatemia, and long-term stimulation of parathyroid hormone secretion, leading to elevation in PTH level and causing SHPT [18]. Due to multiple system and organ lesions, such as systemic itching, pain, pathological fractures, mobility disorders, coagulation disorders, hypoalbuminemia, renal anaemia, etc., there is an undoubtedly elevated risk of surgery and difficulty of perioperative nursing for SHPT patients [19]. Thus, figuring out the most effective nursing process and model for SHPT patients is crucial.

FTS has received successful applications in multiple disciplinary fields [20] [21]. Most reports have validated the effectiveness of FTS, such as shortening hospital stays and mitigating complications [22] [23]. Recent research has affirmed the superiority of the FTS concept. The optimised nursing model is conducive to reducing surgical stress and has an immunoprotective impact. The clinical pathway of FTS nursing avoids blindness and mechanisation of conventional nursing. Dietary guidance is a vital part of the FTS concept [24]. Therefore, food structure intervention needs to develop a food structure table based on the patient's physical condition and treatment characteristics and combine disease situation, especially the advice of a nutritionist, to develop a dietary plan jointly. This research applies food structure intervention based on the FTS concept to perioperative nursing of SHPT patients. The results demonstrated that after the intervention, HGB and ALB levels in OG exhibited elevation relative to those in CG, indicating that food structure intervention based on the FTS concept can improve the nutritional status of SHPT patients. After the intervention, blood calcium level and calcium-phosphorus product in OG exhibited elevation relative to those in CG, while blood phosphorus and iPTH levels in OG exhibited depletion relative to those in CG. This indicates that food structure intervention based on the FTS concept can improve calcium and phosphorus metabolism balance in SHPT patients. This is because food structure intervention facilitates patients' normal diet, timely supplementing protein, vitamins, and calcium absorption, and helping patients balance nutrition and calcium-phosphorus metabolism. Food structure intervention can strengthen communication with patients and their family members, elevate patients' immunity, and ensure sufficient intake and absorption of nutrients.

Yan and colleagues [25] studied the correlation between mineral bone metabolism and CRP in SHPT patients during the perioperative period. They investigated changes in serum calcium, phosphorus, and parathyroid hormone (PTH) levels alongside high-sensitivity C-reactive protein (hs-CRP) before and after surgery, finding a notable reduction in hs-CRP levels post-operation, which was consistent with the findings in our study [25]. Both studies underscored the importance of mineral bone metabolism regulation, such as decreased iPTH and improved calcium-phosphorus balance in the postoperative period. However, while our study focused on the effects of food structure intervention and fast-track surgery on overall recovery and nutritional status, including inflammatory markers like CRP IL-6, and TNF-β, Yan and colleagues [25] zeroed in on PTH-related biomarkers and their direct correlation with inflammation post-surgery. Notably, Yan's research speculated that there may have been an optimal range of PTH concentrations to reduce inflammation, which complemented the broader findings in our study that emphasised nutrition and FTS principles in enhancing postoperative recovery and lowering inflammation.

Asada et al. [26] explored the relationship between calcium and phosphate levels and mortality in SHPT patients, finding that both were associated with increased mortality regardless of iPTH levels. In contrast, our study focused on the impact of food structure intervention in SHPT patients, emphasising improvements in bone metabolism and reduced inflammation post-surgery. While both studies address SHPT-related complications, Asada et al. [26] highlight the risks of abnormal calcium and phosphate levels. In contrast, our research suggests that targeted nutritional interventions can help mitigate these risks by improving mineral metabolism and inflammation.

CRP is not only a non-specific inflammatory marker but also directly participates in inflammation and cardiovascular diseases such as atherosclerosis and is the most potent predictor and risk element of cardiovascular diseases; IL-1 is a vital mediator showing protective response to trauma in the body and is a cytokine upregulating after tissue damage, and the more severe the damage, the higher the serum IL-1 concentration; TGF-β exerts a crucial role in inducing systemic inflammation and immune response after surgery, and different traumas can cause varying degrees of cytokine response, which is a vital indicator of stress in the body [27]. After the intervention, CRP IL-1, and TNF-β levels in OG exhibited depletion relative to those in CG, indicating that food structure intervention based on the FTS concept can mitigate postoperative inflammatory response in SHPT patients. Herein, VAS scores in OG exhibited depletion relative to those in CG, MRC scores in OG exhibited elevation relative to those in CG, and incidence of complications in OG exhibited depletion relative to that in CG, indicating that food structure intervention based on the FTS concept can relieve postoperative pain, elevate postoperative muscle strength, and facilitate postoperative recovery of patients. This is because food structure intervention based on the FTS concept emphasises postoperative scientific diet and reasonable activities to ensure rehabilitation effects.

A limitation of our study was its single-centre design, which may limit the generalizability of the results. Additionally, the absence of long-term follow-up prevented us from assessing the sustained effects of food structure intervention on bone metabolism and inflammation. We also did not conduct a detailed mechanistic analysis of how the intervention interacted with PTH regulation. Furthermore, the study did not account for all potential confounders, such as comorbidities or dialysis regimens. A larger, multicentre trial could have strengthened the external validity of our findings.

## Conclusion

In conclusion, our study shows that food structure intervention based on the fast-track surgery (FTS) concept significantly improves postoperative recovery in SHPT patients. It enhanced nutritional status, balanced mineral metabolism, reduced inflammation, and decreased postoperative pain and complications. These findings support the integration of FTS and tailored nutrition into perioperative care, offering an effective strategy for improving outcomes and reducing complications in SHPT patients.

## Dodatak

### Conflict of interest statement

This study was supported by Medical Science Research Projectof Hebei (No. 20230735). All the authors declare that they have no conflict of interest in this work.

## References

[1] Li D, Guo B, Liang Q, Liu Y, Zhang L, Hu N, Zhang X, Yang F, Ruan C (2020). Tissue-engineered parathyroid gland and its regulatory secretion of parathyroid hormone. Journal of Tissue Engineering and Regenerative Medicine.

[2] Liu Y, Zhang L, Hu N, Shao J, Yang D, Ruan C, Huang S, Wang L, Lu W W, Zhang X, Yang F (2022). An optogenetic approach for regulating human parathyroid hormone secretion. Nature Communications.

[3] Rodriguez-Ortiz M E, Rodriguez M (2020). Recent advances in understanding and managing secondary hyperparathyroidism in chronic kidney disease. F1000 Research.

[4] Chowdhary R, Khan R B, Masarkar N, Malik R, Goel S K (2022). An association of VDR gene polymorphism in hypovitaminosis D mediated secondary hyperparathyroidism in adolescent girlsČ A tertiary hospital study in central India. Steroids.

[5] Levy A R, Xing S, Brunelli S M, Cooper K, Finkelstein F O, Germain M J, Kimel M, Platt R W, Belozeroff V (2020). Symptoms of Secondary Hyperparathyroidism in Patients Receiving Maintenance Hemodialysis: A Prospective Cohort Study. Journal of Kidney Diseases: The Official Journal of the National Kidney Foundation.

[6] D.Marco L, Checa-Ros A, Gamero D, Soto C, Salazar J, Nava M, Bermúdez V, Dapena F (2022). Etelcalcetide and Paricalcitol in Chronic Kidney Disease: When the Target Is Inflammation. Healthcare (Basel).

[7] Bozic M, Diaz-Tocados J M, Bermudez-Lopez M, Forné C, Martinez C, Fernandez E, Valdivielso J M (2022). Independent effects of secondary hyperparathyroidism and hyperphosphataemia on chronic kidney disease progression and cardiovascular events: An analysis from the NEFRONA cohort. Nephrology, Dialysis, Transplantation: Official Publication of the European Dialysis and Transplant Association - European Renal Association.

[8] Chen C, Chen N, Wu F, Wu M (2020). Impact of denosumab on cardiovascular calcification in patients with secondary hyperparathyroidism undergoing dialysis: A pilot study. Osteoporosis International: A Journal Established as Result of Cooperation Between the European Foundation for Osteoporosis and the National Osteoporosis Foundation of the USA.

[9] Gong W, Xie X, Lin X, Meng Z, Wang Y (2021). Risk factors affecting graft survival after parathyroidectomy and parathyroid autotransplantation in patients on maintenance hemodialysis. Nan Fang Yi Ke Da Xue Xue Bao = Journal of Southern Medical University.

[10] Sari R, Yabanoglu H, Hargura A, Kus M, Arer I (2020). Outcomes of Total Parathyroidectomy with Autotransplantation versus Subtotal Parathyroidectomy Techniques for Secondary Hyperparathyroidism in Chronic Renal Failure. Journal of the College of Physicians and Surgeons-Pakistan: JCPSP.

[11] He H, Yang G, Wang S, Han X, Li J (2022). Fast-track surgery nursing intervention in CRC patients with laparotomy and laparoscopic surgery. Medicine (Baltimore).

[12] Wu X, Xu M, Ma Y (2020). Fast-track surgery in single-hole thoracoscopic radical resection of lung cancer. Journal of BUON: Official Journal of the Balkan Union of Oncology.

[13] Wang W, Liu F, Zhang Q, Jiang G, Zheng H, Zhang W (2022). Effect of high-quality nursing on orthopedic trauma based on a fast-track surgery model. American Journal of Translational Research.

[14] Maurer T, Belau M H, von Grundherr J, Schlemmer Z, Patra S, Becher H, Schulz K, Zyriax B, Schmalfeldt B, Chang-Claude J (2022). Randomised controlled trial testing the feasibility of an exercise and nutrition intervention for patients with ovarian cancer during and after first-line chemotherapy (BENITA-study). BMJ Open.

[15] Lewis J D, Sandler R S, Brotherton C, Brensinger C, Li H, Kappelman M D, Daniel S G, Bittinger K, Albenberg L, Valentine J F, Hanson J S, Suskind D L, Meyer A, Compher C W (2021). A Randomized Trial Comparing the Specific Carbohydrate Diet to a Mediterranean Diet in Adults With Crohn's Disease. Gastroenterology.

[16] Luyao H, Xiaoxiao Y, Tianxiao F, Yuandong L, Ping W (2022). Management of Cervical Spondylotic Radiculopathy: A Systematic review. Global Spine Journal.

[17] Schreiber-Katz O, Siegler H A, Wieselmann G, Kumpe M, Ranxha G, Petri S, Osmanovic A (2023). Improvement of muscle strength in specific muscular regions in nusinersen-treated adult patients with 5q-spinal muscular atrophy. Scientific Reports.

[18] Bover J, Gunnarsson J, Csomor P, Kaiser E, Cianciolo G, Lauppe R (2021). Impact of nutritional vitamin D supplementation on parathyroid hormone and 25-hydroxyvitamin D levels in non-dialysis chronic kidney disease: A meta-analysis. Clinical Kidney Journal.

[19] Bellone F, Cinquegrani M, Nicotera R, Carullo N, Casarella A, Presta P, Andreucci M, Squadrito G, Mandraffino G, Prunestì M, Vocca C, de Sarro G, Bolignano D (2022). Role of Vitamin K in Chronic Kidney Disease: A Focus on Bone and Cardiovascular Health. International Journal of Molecular Sciences.

[20] Grifka J, Greimel B, Maderbacher G (2022). Day-case outpatient endoprosthetics - 'Ultra-Fast-Track'. Der Orthopade.

[21] Fazzalari A, Srinivas S, Panjwani S, Pozzi N, Friedrich A, Sheoran R, Sabato J, Durocher D, Reznek M, Aiello F, Litwin D, Cahan M A (2021). A Fast-Track Pathway for Emergency General Surgery at an Academic Medical Center. Journal of Surgical Research.

[22] Maderbacher G, Meyer M, Grifka J, Holzapfel D, Greimel F (2022). Results and lessons learned in fast-track arthroplasty. Der Orthopade.

[23] Goodson K M, Kee J R, Edwards P K, Novack A J, Stambough J B, Siegel E R, Barnes C, Mears S C (2020). Streamlining Hospital Treatment of Prosthetic Joint Infection. Journal of Arthroplasty.

[24] McCarthy C, Fletcher N (2020). Early Extubation in Enhanced Recovery from Cardiac Surgery. Critical Care Clinics.

[25] Yan L, Xiong Q, Xu Q, Ren P, Li T, Cao H, Shao F (2023). Study on the correlation between mineral bone metabolism and CRP in patients with SHPT during perioperative period. Immun Inflamm Dis.

[26] Asada S, Yokoyama K, Miyakoshi C, Fukuma S, Endo Y, Wada M, Nomura T, Onishi Y, Fukagawa M, Fukuhara S, Akizawa T (2020). Relationship between serum calcium or phosphate levels and mortality stratified by parathyroid hormone level: An analysis from the MBD-5D study. Clin Exp Nephrol.

[27] Zhao J, Zhang L, Liu Y, Cheng Q (2021). Effect of Shexiang Baoxin Pill (麝香保心丸) in Alleviating Early Hypertensive Renal Injury in Rats. Chinese Journal of Integrative Medicine.

